# Genome-Wide Association Study for Indicator Traits of Sexual Precocity in Nellore Cattle

**DOI:** 10.1371/journal.pone.0159502

**Published:** 2016-08-05

**Authors:** Natalia Irano, Gregório Miguel Ferreira de Camargo, Raphael Bermal Costa, Ana Paula Nascimento Terakado, Ana Fabrícia Braga Magalhães, Rafael Medeiros de Oliveira Silva, Marina Mortati Dias, Annaiza Braga Bignardi, Fernando Baldi, Roberto Carvalheiro, Henrique Nunes de Oliveira, Lucia Galvão de Albuquerque

**Affiliations:** 1 Departamento de Zootecnia, Faculdade de Ciências Agrárias e Veterinárias, Universidade Estadual Paulista, Jaboticabal, São Paulo, Brasil; 2 Grupo de Melhoramento Animal de Mato Grosso, Instituto de Ciências Agrárias e Tecnológicas, Universidade Federal de Mato Grosso, Rondonópolis, Mato Grosso, Brasil; China Agricultrual University, CHINA

## Abstract

The objective of this study was to perform a genome-wide association study (GWAS) to detect chromosome regions associated with indicator traits of sexual precocity in Nellore cattle. Data from Nellore animals belonging to farms which participate in the DeltaGen^®^ and Paint^®^ animal breeding programs, were used. The traits used in this study were the occurrence of early pregnancy (EP) and scrotal circumference (SC). Data from 72,675 females and 83,911 males with phenotypes were used; of these, 1,770 females and 1,680 males were genotyped. The SNP effects were estimated with a single-step procedure (WssGBLUP) and the observed phenotypes were used as dependent variables. All animals with available genotypes and phenotypes, in addition to those with only phenotypic information, were used. A single-trait animal model was applied to predict breeding values and the solutions of SNP effects were obtained from these breeding values. The results of GWAS are reported as the proportion of variance explained by windows with 150 adjacent SNPs. The 10 windows that explained the highest proportion of variance were identified. The results of this study indicate the polygenic nature of EP and SC, demonstrating that the indicator traits of sexual precocity studied here are probably controlled by many genes, including some of moderate effect. The 10 windows with large effects obtained for EP are located on chromosomes 5, 6, 7, 14, 18, 21 and 27, and together explained 7.91% of the total genetic variance. For SC, these windows are located on chromosomes 4, 8, 11, 13, 14, 19, 22 and 23, explaining 6.78% of total variance. GWAS permitted to identify chromosome regions associated with EP and SC. The identification of these regions contributes to a better understanding and evaluation of these traits, and permits to indicate candidate genes for future investigation of causal mutations.

## Introduction

Reproductive traits, including female sexual precocity, are directly associated with the profitability of beef cattle production systems. [1], studying economic values for reproductive traits, found that these traits can be up to 13 times more important than growth traits. Age at puberty of females is an economically important trait, especially in *Bos taurus indicus* animals that are sexually less precocious than *Bos taurus taurus* [2,3]. However, selection for this trait is difficult since the identification of the onset of puberty in heifers, i.e., the age when the female expresses her reproductive capacity [4], requires the adaptation of management, including the use of a teaser bull or hormone tests for the detection of heat, and consequently increases the cost of the production system. On the other hand, indicator traits of sexual precocity, such as the occurrence of early pregnancy of heifers and scrotal circumference, are obtained more easily in the herd since they are part of routine data collection and can therefore be used as selection criteria.

Although these traits can be easily measured in a large number of animals and show high heritability [5–8], they are sex-limited traits. Additionally, in the case of the occurrence of pregnancy, the trait is measured only after first calving. The evaluation of bulls for this trait depends on progeny testing, and accurate estimated breeding values (EBV) for these animals will only become available long after they have been selected [9]. These evaluations are costly and increase the generation interval, reducing annual genetic gain.

Recent technological advances have permitted the use of dense single nucleotide polymorphism (SNP) panels for genome-wide association studies (GWAS). In this respect, SNPs associated with quantitative trait loci (QTL) that influence the expression of traits can be identified and used for the construction of SNP panels, which will help with the selection process [10]. According to [11], one approach to improve the accuracy of genomic predictions for fertility is the incorporation of SNP panels associated with genes that influence reproductive traits.

Among the methods used for GWAS, the classical method is based on testing a single marker at a time, in which each SNP is treated as a covariate in the model [12]. Although this method is advantageous in terms of the ease of use of significance tests, it is likely to result in poor fit of the data compared to methods that consider all SNPs together [13]. These methods, in turn, are performed in multiple-steps in which EBVs are estimated using the traditional BLUP model and these EBVs will then be used as pseudo-phenotypes in genomic prediction models to estimate marker effects [14,15]. In these procedures, only animals with known phenotype and genotype are included in the analysis.

Single-step GBLUP (ssGBLUP), which combines pedigree, phenotype and genotype data, was proposed by [16]. According to [13], ssGBLUP is based on an infinite model that assumes equal variance for SNP effects. This fact limits the use of this method since it does not reflect the real situation for all traits of economic interest. These authors therefore proposed weighted ssGBLUP (WssGBLUP), which combines pedigree, phenotype and genotype data, and different weights are attributed to the markers in an iterative process to update the SNP solutions.

Using GWAS, some genes associated with reproductive traits in cattle have been described in the literature for Zebu animals [9,17–21]. However, few studies are available for indicator traits of sexual precocity in Nellore cattle.

The objective of this study was to perform GWAS to detect chromosome regions associated with indicator traits of sexual precocity in Nellore cattle using a single-step method. This study will provide data for a better understanding of the distribution of genes that affect these traits and for genomic selection.

## Materials and Methods

### Phenotypic data

Data from Nellore animals belonging to eight farms, which participate in the DeltaGen^®^ and Paint^®^ (CRV Lagoa) breeding programs, were used. Of these farms, six are located in the Midwest (in the states of Goiás, Mato Grosso and Mato Grosso do Sul), one in Southeast (in the state of São Paulo) and one in Northeast (in the state of Bahia) of Brazil.

The traits associated with sexual precocity used in this study were occurrence of early pregnancy of heifers (EP) and scrotal circumference (SC). EP was defined based on the conception and calving of the heifer as long as the animal had entered the breeding season at about 16 months of age. A value of 1 (success) was attributed to heifers that calved at less than 31 months of age and a value of 0 (failure) to heifers that failed, i.e., animals that calved after 31 months of age. SC was measured at yearling in centimeters.

The fixed effects considered for the formation of the contemporary groups (CG) for the two traits included year of birth, farm, and management group at birth, weaning and yearling. Contemporary groups showing no variability in EP, i.e., those in which all animals had the same response category, and CG with records outside the interval given by the mean of the group plus or minus three standard deviations for SC were eliminated. Additionally, for both traits, CG with fewer than four observations were excluded from the analysis.

For the two traits, the model included additive genetic and residual as random effects and CG as fixed effect. For SC, the linear effect of age of animal at recording was included in the model as a covariate. Table 1 shows the number of animals used in the analysis and the results of descriptive statistics of the traits studied. Phenotypic data of 73,359 females and 87,612 males, for EP and SC, respectively, were used in the analysis. The relationship matrix contained 203,017 animals.

**Table 1:** Statistics, heritability and average accuracy of the estimated breeding values of the indicator traits of sexual precocity in Nellore cattle.

Trait	N_animal_	Mean	Minimum	Maximum	h^2^	acc
EP (%)	73,359	18.6*	--	--	0.30	0,60
SC (cm)	87,612	26.75	15	40	0.41	0,66

*Represented in percentage;

N_animal_, number of animals in genomic association analysis; h^2^, heritability; acc, average accuracy of the estimated breeding values; EP, occurrence of early pregnancy; SC, scrotal circumference.

### Genotypic data

The genotype data from 1,770 females, born between 2007 and 2009, and from 1,680 males, born between 1993 and 2012, were used. Additionally, the genotype data from 611 sires (father of the females and males used in this study), born between 1965 and 2006, were used; of these, 223 had phenotypic information for SC. Except for part of the sires, performance records and pedigree information were available for all genotyped animals. The animals were genotyped using the high-density Illumina Bovine HD Assay (Illumina, Inc., San Diego, CA, USA), which contains 777,962 SNPs.

Quality control of the genotype data was performed in an iterative process, in which first SNPs and then samples were excluded at each iteration until none of the SNPs or samples was excluded. The following criteria were used for the exclusion of SNPs: non-autosomal regions; mapped at the same position; p-value for Hardy-Weinberg equilibrium less than 10^−5^; minor allele frequency less than 2%; GenCall score less than 15%; and genotyping efficiency of each SNP (call frequency) less than 95%. Samples with a call rate less than 90% and/or duplicate samples were excluded. After quality control, 412,993 SNPs remained for analysis.

### Data analysis

The SNP effects were estimated using the weighted single-step method (WssGBLUP) proposed by [13]. The observed phenotypes of EP and SC were used as dependent variables. All animals with available genotypes and phenotypes, in addition to those with only phenotype information, were used. A single-trait animal model was applied to predict the breeding values:

$$y=Xb+Z_{a}a+e$$ (1)

where *y* is the vector of observed phenotypes for genotyped and non-genotyped animals; ***X*** is the incidence matrix of fixed effects; *b* is the vector of fixed effects, including CG and age at recording as the covariate for SC; ***Z***_***a***_ is the incidence matrix of additive genetic effects; *a* is the random vector of additive genetic effects; and *e* is the vector of residual effects. A threshold model was assumed for EP, which relates the phenotype observed on a categorical scale to an underlying continuous normal scale.

For genetic effects, it was assumed that $a\sim N\left(0,H\sigma_{a}^{2}\right)$, where ***H*** is the relationship matrix based on both genomic information and pedigree information and σ^*2*^_*a*_ is the additive genetic variance. For residual effects, it was assumed that $e\sim N\left(0,I\sigma_{e}^{2}\right)$, where ***I*** is an identity matrix and σ^*2*^_*e*_ is the residual variance. Since σ^*2*^_*e*_ cannot be estimated on the underlying scale [22], a value of 1 was attributed to σ^*2*^_*e*_ for EP. The inverse of matrix ***H*** can be written as follows [23]:

$$H^{-1}=A^{-1}+\left[\begin{array}{cc} 0 & 0\\ 0 & G^{-1}-A_{22}^{-1} \end{array}\right]$$ (2)

where ***A*** is the numerator relationship matrix based on pedigree for all animals; ***A***_***22***_ is the numerator relationship matrix based on pedigree for genotyped animals only; and ***G*** is the genomic relationship matrix for genotyped animals calculated according to [14]:

G=ZDZ′λ (3)

where ***Z*** is the matrix obtained by subtracting matrix ***P***, which contains the allele frequency of the second allele, from genotype matrix ***M***, which its dimension is the number of individuals (*n*) by number of loci (*m*); ***D*** is a diagonal matrix of weights for SNP variances; and λ is a ratio of variances or a normalizing constant [14]:

$$\lambda =\frac{\sigma_{u}^{2}}{\sigma_{a}^{2}}=\frac{1}{\sum_{i=1}^{M}2p_{i}\left(1-p_{i}\right)}$$ (4)

where *M* is the number of SNPs and *p*_*i*_ is the allele frequency of the second allele of marker *i*.

The solutions of the SNP effects (*û*) were obtained from the breeding values using the following algorithm of scenario S1 [13]:

1. In the first iteration ***D***_(*t*)_ *=* ***I***, ***G***_(*t*)_ *=* ***ZD***_(*t*)_***Z′***λ, where *t* is the number of iterations.

2. Calculate the breeding value of all animals using ssGBLUP.

3. Calculate *${û}_{\left(t\right)}=\lambda D_{\left(t\right)}Z\prime G_{\left(t\right)}^{-1}{â}_{g}$*, where *â*_*g*_ is the breeding value of genotyped animals.

4. Calculate weights $d_{i\left(t+1\right)}={û}_{i\left(t\right)}^{2}2p_{i}\left(1-p_{i}\right)$ for each SNP [24].

5. Normalize the weights so that the additive genetic variance remains constant: $D_{\left(t+1\right)}=\frac{tr\left(D_{\left(0\right)}\right)}{tr\left(D_{\left(t+1\right)}\right)}D_{\left(t+1\right)}$.

6. Calculate ***G***_(*t+1*)_ *=* ***ZD***_(*t+1*)_***Z′***λ.

7. *t* = *t* + 1.

8. Exit or go back to step 3.

Thus, matrix ***G*** was recalculated for the prediction of SNP effects at each iteration, while *â*_*g*_ was obtained only once and was not altered during the iterations. Three iterations were performed in total.

The programs of the BLUPF90 family [25] were used for WssGBLUP analysis. A Bayesian approach was used for the analysis of EP, which consisted of a single chain of 300,000 cycles, with a conservative burn-in period of 30,000 cycles and a thinning interval of 30 cycles. Thus, 9,000 samples were effectively used.

The results of GWAS are reported as the proportion of variance explained by windows of 150 adjacent SNPs. For the two traits, the 10 windows that explained the highest proportion of variance were identified. For EP, these windows ranged from 0.58 (chromosome 27) to 3.02 Mb (chromosome 21), with an average of 1.04 Mb. For SC, these windows ranged from 0.52 (chromosome 19) to 1.91 Mb (chromosome 13), with an average of 0.93 Mb.

The NCBI’s Map Viewer tool for the bovine genome was used for identification of the genes using the UMD 3.1 assembly as the reference map (http://www.ncbi.nlm.nih.gov/projects/mapview/map_search.cgi?taxid=9913&build=104.0). Only genes present within the windows of 150 SNPs were considered. A manual search was performed to know the metabolic function of the genes identified in order to understand the action of these genes on the traits studied.

## Results and Discussion

**Fig 1:**
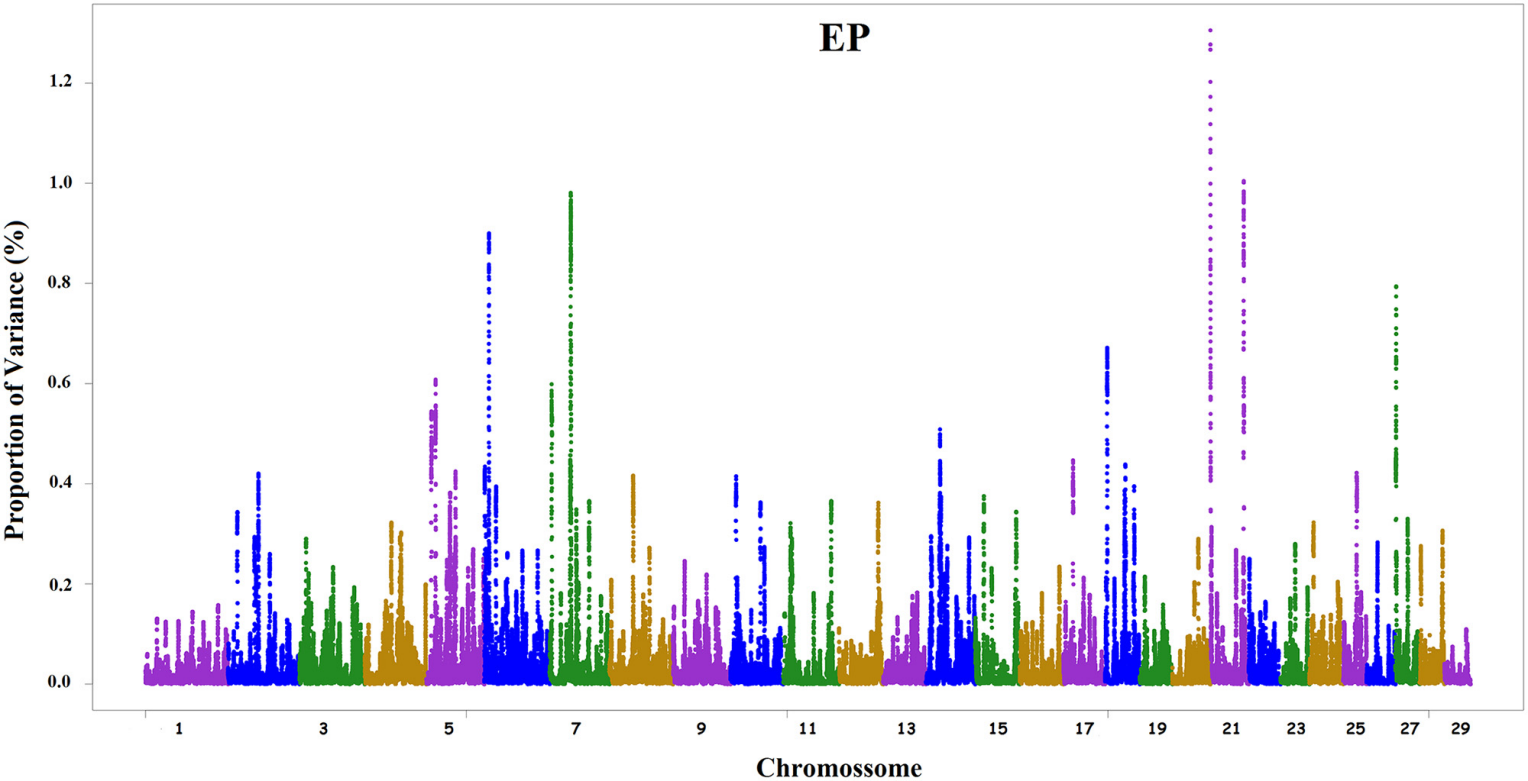
Manhattan plot for the occurrence of early pregnancy (EP) in Nellore cattle. The y-axis shows the proportion of variance explained by the windows of 150 SNPs and the x-axis indicates the chromosome number.

**Fig 2:**
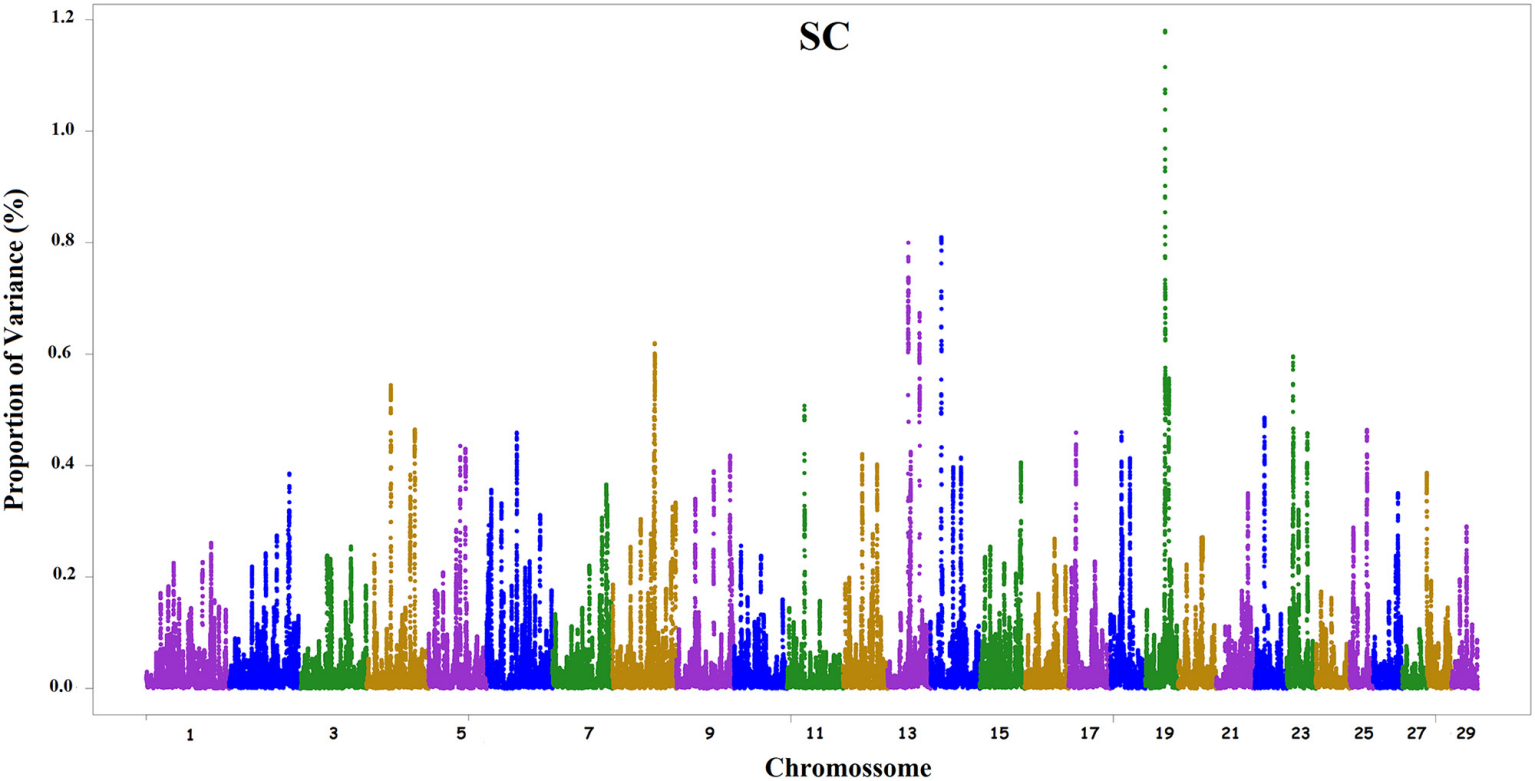
Manhattan plot for scrotal circumference (SC) in Nellore cattle. The y-axis shows the proportion of variance explained by the windows of 150 SNPs and the x-axis indicates the chromosome number.

As observed in the simulation studies of [13] and [21], when SNP weights were recomputed at each iteration and SNP effects were subsequently recalculated, small-effect SNPs had their effects reduced, with effects close to zero, while large-effect SNPs became even more pronounced (data not shown). However, the third iteration revealed a larger number of peaks in different chromosomes. The SNP windows obtained in the second iteration that explained the highest proportion of variance can be observed in Figs 1 and 2, where Manhattan plots are shown for the traits EP and SC, respectively. General information about all results of WssGBLUP for both traits are in S1 File.

In the study of [13] using WssGBLUP, the second iteration provided the highest prediction accuracy of SNP effects. According to the authors, a combination of weights occurred in the second iteration that minimized estimation errors and reflected the reality that SNPs adjacent to a QTL contribute to the estimation of that QTL. Using simulated data, [21] reported that for WssGBLUP, among the three iterations analyzed, the second iteration was the best to detect QTLs accurately. In the third iteration, false-positive signals may have been captured by the markers.

The proportions of variance explained by the windows (Figs 1 and 2) indicate the polygenic nature of EP and SC, i.e., the indicator traits of sexual precocity studied here are probably controlled by a large number of genes, including some of moderate effect. The 10 windows with large effects obtained for EP are located on chromosomes 5, 6, 7, 14, 18, 21 and 27, and together explained 8.91% of the total genetic variance. Chromosome 21 exhibited two large-effect windows for EP, explaining 2.31% of total variance. Table 2 shows known genes found in these windows.

**Table 2:** Identification of genes within the 10 windows of 150 SNPs that explained the highest proportion of genetic variance for the occurrence of early pregnancy in Nellore cattle.

Chr	Position (bp)	Genes	Var (%)
5	8885458–10124194	*LOC104972336*, *LOC104972335*, *SYT1*, *LOC104972334*, *PAWR*, *LOC104972337*, *LOC104972338*, *PPP1R12A*, *OTOGL*, *LOC616702*, *LOC100139469*, *LOC104972340*, *LOC104972339*, *LOC100849027*, *PTPRQ*	0.54
	16060262–17119004	*MGAT4C*, *LOC101906434*, *LOC781871*	0.61
6	10646200–11661005	*LOC781502*, *LOC101907884*, *LOC104972659*, *NDST4*	0.90
7	3116169–3849099	*LOC104969038*, *PRSS38*, *LOC100138519*, *LOC784059*, *SNAP47*, *JMJD4*, *ZNF354B*, *VN2R401P*, *VN2R402P*, *VN2R403P*, *LOC104969039*, *LOC100295355*, *ATP13A1*, *GMIP*, *LPAR2*, *PBX4*, *LOC104969040*, *CILP2*, *YJEFN3*, *NDUFA13*, *TSSK6*, *GATAD2A*	0.60
	41289319–42032123	*MGAT1*, *LOC104969159*, *ZFP62*, *LOC618125*, *LOC614895*, *LOC104972830*, *LOC530231*, *OR2V1*, *LOC100299289*, *LOC101902906*, *LOC104972831*, *BTNL9*, *OR2V2*, *LOC508420*, *LOC104969160*, *TRNAL-AAG*, *TRNAT-UGU*, *TRIM7*, *TRNAA-UGC*, *TRNAK-CUU*, *TRNAV-AAC*, *TRNAV-CAC*, *TRIM41*, *GNB2L1*, *TRIM52*, *LOC509006*, *IFI47*, *ZNF496*, *NLRP3*	0.98
14	22610144–23399257	*LOC104974016*, *PCMTD1*, *LOC101906226*, *LOC104974020*, *ST18*, *LOC100141260*, *LOC101906592*, *FAM150A*, *RB1CC1*, *LOC104974017*, *NPBWR1*, *OPRK1*	0.51
18	4263730–4907873	*LOC104974747*, *LOC101905587*, *MON1B*, *LOC104974727*, *SYCE1L*, *ADAMTS18*, *LOC104974729*, *LOC104974728*	0.67
21	8725–3028689	*SNRPN*, *SNURF*, *NDN*, *MAGEL2*, *MKRN3*, *LOC789575*, *LOC100336464*, *LOC101908683*, *LOC101904983*, *LOC789520*, *LOC104968504*, *LOC101905258*, *LOC101905210*, *LOC619073*, *LOC789547*, *LOC101905329*, *LOC104975302*, *LOC100139608*, *LOC615561*, *LOC101906493*, *LOC101905769*, *LOC101909016*, *LOC101902420*, *LOC101906577*, *LOC101909328*, *LOC100848941*, *LOC101907203*, *UBE3A*, *LOC100140958*, *ATP10A*	1.31
	61928582–62528341	*SYNE3*, *LOC100847284*, *GLRX5*, *LOC104975438*, *TCL1B*, *TCL1A*, *TUNAR*, *C21H14orf132*	1.00
27	992755–1574730	*CSMD1*, *LOC104976023*	0.79

Chr, chromosome; Var, additive genetic variance explained by the window.

The present study identified windows associated with EP that had been previously associated with age at first calving (Chr05/16.06–17.12Mb and Chr07/41.29–42.03Mb) and heifer reconception (Chr14/22.61–23.40Mb) by [21] using real data (the same database and methodological criterion as employed in this study). These coinciding results were expected since both EP and age at first calving are indicator traits of female sexual precocity and exhibit a favorable genetic correlation [8].

Some of the genes found in these regions are important because they act as olfactory receptors, including the genes of window Chr07/41.29–42.03Mb (*LOC618125*, *LOC614895*, *LOC530231*, *OR2V1*, *LOC100299289*, *OR2V2*, and *LOC508420*), in addition to *LOC100139608* located in window Chr21/0.01–3.02Mb. A physiological explanation exists for the association of these genes with EP, since olfactory receptors have a function in germ cells, which are the precursors of gametes [26], affecting their production and consequent reproduction. Olfactory receptors are also expressed in the gametes themselves where they play a role in chemotaxis between the sperm and egg to promote fertilization of the gametes whose major histocompatibility complex (MHC) genes are the most diverse [27], guaranteeing the success of fertilization.

Some of the genes found in this window of chromosome 7 have been identified in studies analyzing selection signatures in different cattle breeds, such as the *MGAT1* gene [28] involved in the production of gametes [29], or have been detected in fine mapping studies, such as the *ZNF496* gene associated with fertility in dairy cattle [30]. One group of genes identified in another window of chromosome 7 (Chr07/3.12–3.85Mb) act as vomeronasal receptors (*VN2R401P*, *VN2R402P*, and *VN2R403P*). Expressed in the vomeronasal organ of mammals, the function of these receptors is to detect pheromones related to reproduction and the selection of sexual partners [31], guaranteeing offspring immunity.

The Chr21/0.01–3.02Mb region is under the epigenetic effect of methylation and comprises the *SNRPN*, *SNURF*, *NDN*, *MAGEL2* and *UBE3A* genes. These genes exert a known function in the implantation of embryos in different species. In this respect, failed implantation of the embryo may be caused by imprinting defects or by the fact that these genes are expressed differentially during the process of implantation [32]. Furthermore, the *MAGEL2* gene has been reported to be essential for placental development in pigs [33] and is associated with SC in Nellore cattle [34].

Other genes are important because of their biological role or because they are expressed in target tissues for reproduction. The expression of the *PAWR* gene (Chr05/8.89–10.12Mb) is altered in the preovulatory follicle of buffaloes [35] and the *LPAR2* gene (Chr07/3.2–3.85Mb) is important for the development of pregnancy in cattle, exerting an effect on the corpus luteum [36]. The *GNB2L1* (Chr07/41.29–42.03Mb) and *MON1B* (Chr18/4.26–4.91Mb) genes are involved in embryo development and may be directly associated with the pregnancy rate of females and with maintenance of the conceptus during the early stage [11,33]. The *LOC101905587* gene (*TIMP1*), which is also located on chromosome 18, participates in follicular development in cattle [37], as well as in endometrial remodeling [38]. Another gene identified is the *RB1CC1* gene (Chr14/22.61–23.40Mb), which participates in meiosis and is associated with premature ovarian failure in women [39].

The window found on chromosome 14 is located between the 20 and 30 Mb regions. Some studies have identified SNPs in these regions that are associated with various traits in different cattle breeds: age at puberty in males and females, postpartum anestrus interval, calving ease, rump height, height, body size, stillbirth rate, growth hormone levels (IGF-1), weight, and fat deposition [9,17,40–44].

Some of the regions identified here have also been detected in other studies investigating reproductive traits in cattle, such as regions Chr05/8.89–10.12Mb [45–47], Chr07/3.12–3.85Mb and Chr07/41.29–42.03Mb [46], and Chr21/0.01–3.02Mb [46,47]. Taken together, these findings demonstrate that cattle breeds share regions that exert a great influence on reproductive traits.

For SC, the large-effect windows are located on chromosomes 4, 8, 11, 13, 14, 19, 22 and 23, explaining 6.78% of the total genetic variance. Table 3 shows the genes identified for SC. Among these genes, the *OVOL2* gene (Chr13/37.83–38.93Mb) is important since it is highly expressed in the testes and during spermatogenesis and, at lower levels, in the ovaries of rats [48]. [49] detected the expression of this regulatory gene in male germ cells of humans.

**Table 3:** Identification of genes within the 10 windows of 150 SNPs that explained the highest proportion of genetic variance for scrotal circumference in Nellore cattle.

Chr	Position (bp)	Genes	Var (%)
4	48976782–49824079	*SLC26A4*, *LOC104972047*, *CBLL1*, *SLC26A3*, *TRNASTOP-UCA*, *DLD*, *LOC101906258*, *LAMB1*, *MIR2418*, *NRCAM*, *LOC104972048*	0.54
8	73907982–75004202	*TRNASTOP-CUA*, *EBF2*, *MIR2898*, *MIR2404-2*, *LOC101907345*, *TRNAW-CCA*, *LOC104969415*, *TRNAG-CCC*, *PPP2R2A*, *LOC104969416*, *BNIP3L*	0.62
11	29596527–30373422	*EPCAM*, *MSH2*, *KCNK12*, *TRNAG-UCC*, *LOC104973335*, *LOC786908*, *LOC104973336*, *MSH6*, *FBXO11*, *LOC100300972*, *LOC104933337*	0.51
13	37826545–38932531	*PCSK2*, *LOC104973771*, *SLC6A9*, *LOC101904180*, *LOC104973772*, *LOC100335935*, *BFSP1*, *DSTN*, *RRBP1*, *BANF2*, *LOC104973773*, *LOC104973774*, *SNX5*, *MGME1*, *OVOL2*, *LOC104973775*, *CSRP2BP*, *ZNF133*, *LOC101904281*, *LOC101906934*, *LOC539166*, *DZANK1*, *POLR3F*, *SEC23B*, *LOC100295268*, *DTD1*	0.80
	63267570–65181056	*BPIFB1*, *BPIFB5*, *CDK5RAP1*, *SNTA1*, *CBFA2T2*, *NECAB3*, *C13H20orf144*, *ACTL10*, *E2F1*, *PXMP4*, *ZNF341*, *CHMP4B*, *RALY*, *EIF2S2*, *ASIP*, *AHCY*, *ITCH*, *DYNLRB1*, *MAP1LC3A*, *PIGU*, *TP53INP2*, *NCOA6*, *GGT7*, *ACSS2*, *GSS*, *MYH7B*, *TRPC4AP*, *EDEM2*, *PROCR*, *MMP24*	0.67
14	17826493–18510261	*KLHL38*, *LOC614414*, *LOC100848930*, *FBXO32*, *WDYHV1*, *ATAD2*, *ZHX1*, *C14H8orf76*, *FAM83A*, *TRNAM-CAU*, *LOC104974006*, *TBC1D31*, *DERL1*	0.81
19	36909118–37520214	*LOC104975061*, *LRRC59*, *EME1*, *MRPL27*, *XYLT2*, *TMEM92*, *LOC104975062*, *LOC618012*, *COL1A1*, *HILS1*, *SGCA*, *PPP1R9B*, *PDK2*, *ITGA3*, *DLX3*, *DLX4*, *LOC100196902*, *LOC104975063*, *LOC104975067*, *LOC104975066*, *LOC104975065*, *LOC104975064*, *TAC4*, *KAT7*, *LOC101902801*, *FAM117A*	1.18
	48689556–49207130	*DDX42*, *FTSJ3*, *PSMC5*, *SMARCD2*, *TCAM1*, *GH1*, *CD79B*, *SCN4A*, *PRR29*, *ICAM2*, *LOC506088*, *LOC616254*, *LOC100140873*, *TRNAG-UCC*, *TEX2*, *LOC104975109*, *LOC101902037*, *LOC104975110*, *PECAM1*	0.56
22	16978077–17986079	*TADA3*, *LOC104975513*, *CAMK1*, *OGG1*, *BRPF1*, *CPNE9*, *MTMR14*, *LOC101907007*, *LHFPL4*, *SETD5*, *THUMPD3*, *LOC104975514*, *SRGAP3*, *LOC104975515*, *RAD18*, *CAV3*, *OXTR*, *SSUH2*, *LMCD1*	0.49
23	14017403–14741325	*LOC104969558*, *LOC104969567*, *LOC104969575*, *LOC100847495*, *LOC104969576*, *LRFN2*, *LOC101907037*, *TRNAI-UAU*	0.60

Chr, chromosome; Var, additive genetic variance explained by the window.

Several other genes related to testicular growth and development were identified. Many of these genes are also directly involved in spermatogenesis, such as the *HILS1* gene that remodels chromatin during gamete production [50], the *KAT7* and *ICAM2* genes expressed in Sertoli cells [51,52], the *TCAM1* gene expressed in spermatocytes [53], the *SLC26A3* gene that is essential for sperm capacitation [54], and the *RAD18* gene that plays a crucial role in genome maintenance during sperm production [55]. Other important genes are the *FBXO32* (Chr14/17.83–18.51Mb) and *EBF2* (Chr08/73.91-75Mb) genes related to infertility and hypogonadism in men, respectively [56,57], and the *GH1* gene (Chr19/48.69–49.21Mb) associated with seminal characteristics and sexual behavior in cattle [58].

The window of chromosome 14 associated with SC has also been described by [34] in a study using the same breed and trait, and by [17] who investigated age at SC of 26 cm in Brahman bulls. However, many of the large-effect regions found in the present study were different from those described by [34]. This fact might be explained by the use of different methods and SNP quality control criteria, as well as by the genetic constitution of the population and number of animals used in the study.

Between the two windows observed in this study on chromosome 19, closer to window Chr19/36.91–37.52Mb, [17] detected the *IGFBP4* gene, which is associated with age at puberty in Brahman cattle. The same gene has been associated with heifer conception and postpartum anestrus [9,59]. [60] found two QTLs on chromosome 23 in the same region as observed in the present study, which affect the percentage of viable sperm with an intact cytoplasmic membrane and sperm concentration. [18], evaluating age at first calving and heifer reconception, identified two regions associated with these traits on chromosomes 8 and 13, respectively, close to the regions associated with SC in the present study.

It should be noted that part of the region identified here on chromosome 14 associated with EP has been described in a study investigating meat tenderness in Nellore cattle [61]. Furthermore, the region of chromosome 13 associated with SC has previously been associated with fat thickness also in Nellore cattle [62]. These findings suggest that these regions exert pleiotropic effects, considering the association found here with indicator traits of sexual precocity.

The windows with large effects found for the two traits were not overlapping, although a moderate genetic correlation between these traits exists in Nellore cattle [63]. This fact has also been observed by [64] in a study on causal mutations, in which highly significant SNPs for andrological traits were not significant for female reproductive traits. This suggests that the genetic correlation between male and female reproductive traits is due to small-effect genes that contribute to both traits and/or genes with a larger effect on one trait and a smaller effect on the other. Chromosome binding may also occur since, as observed for the windows cited above, there are genes in the windows associated with SC that exert functions in female reproduction (*ITGA3* –[65]; *DLX3* –[66]; *FAM117A* –[67]; *DERL1* –[68]; *E2F1* –[69]; *MAP1LC3A* –[70]; *LRFN2* –[71]), and there are genes in the windows associated with EP that are involved in male fertility (*TSSK6* –[72]).

## Conclusions

GWAS permitted to identify chromosome regions associated with EP and SC. The identification of these regions contributes to a better understanding and evaluation of these traits, and permits to indicate candidate genes for future investigation of causal mutations.

## Supporting Information

S1 File Percentage of variances. Results of WssGBLUP for occurrence of early pregnancy and scrotal circumference.
(XLSX)
